# 3-(Sulfamic acid)-propyltriethoxysilane on biochar nanoparticles as a practical, biocompatible, recyclable and chemoselective nanocatalyst in organic reactions†

**DOI:** 10.1039/d4ra02265c

**Published:** 2024-07-12

**Authors:** Norolhoda Emad-Abbas, Jalil Naji, Parisa Moradi, Tavan Kikhavani

**Affiliations:** a Department of Physics, Faculty of Science, Ilam University Ilam Iran j.naji@ilam.ac.ir; b Department of Chemistry, Faculty of Science, Ilam University P.O. Box 69315516 Ilam Iran; c Department of Chemical Engineering, Faculty of Engineering, Ilam University Ilam Iran

## Abstract

Recyclable and inexpensive catalysts, waste regeneration, use of available and safe solvents are important principles of green chemistry. Therefore, in this project, biochar nanoparticles (BNPs) were synthesized by the pyrolysis method from chicken manure. Then, 3-(sulfamic acid)-propyltriethoxysilane (SAPES) was immobilized on the surface of BNPs (SAPES@BNPs). The prepared catalyst (SAPES@BNPs) was used as a commercial, practical, biocompatible and reusable catalyst in the selective oxidation of sulfides to sulfoxides. Further, the catalytic application of SAPES@BNPs was explored in the multicomponent synthesis of tetrahydrobenzo[*b*]pyrans under mild and green conditions. BNPs were characterized using SEM, TGA and XRD techniques. SAPES@BNPs were characterized using SEM, FT-IR spectroscopy, WDX, EDS, TGA, and XRD techniques. Particle size distribution was obtained by histogram graph. SAPES@BNPs can be recovered and reused several times. The purity of the products was studied using NMR spectroscopy.

## Abstract 2

Biochar nanoparticles were functionalized by 3-(sulfamic acid)-propyltriethoxysilane (SAPES) as nanocatalyst in the synthesis of sulfoxides and tetrahydrobenzo[*b*]pyrans.

## Graphical abstract

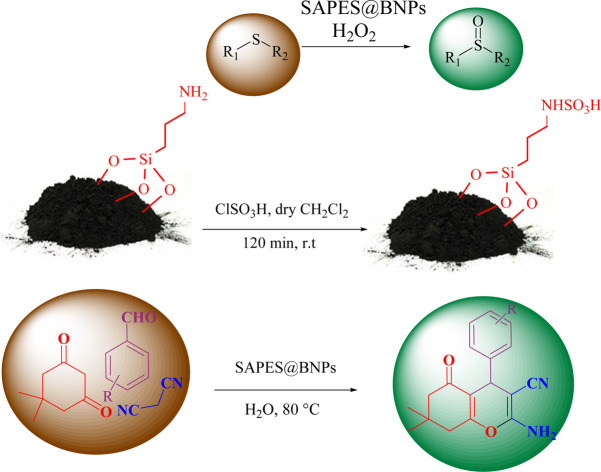

## 1. Introduction

The interaction between starting materials and catalyst species is quick and easy in a homogeneous system because the catalyst particles and the reactants are in one phase, which leads to increases in the catalytic activity of the homogeneous catalysts. However, catalyst recovery and recycling at the end of the reaction is time-consuming and very difficult, and the isolation of the pure products is very difficult.^1–7^ Therefore, the application of homogeneous catalysts is limited despite their high catalytic activity. Whereas, heterogeneous catalysts have different physical phases than reaction media and as they are not soluble in the reaction mixture, they can be easily recovered and reused.^8–11^ Therefore, heterogeneous catalysts have great and important advantages such as easy separation, recyclability, environmental compatibility, and excellent purity of the final product free of catalyst contaminants.^12–15^ In addition, homogeneous acid catalysts lead to equipment corrosion, whereas heterogeneous or immobilized acid catalysts are safe. However, heterogeneous catalysts are less efficient and selective than many homogeneous catalysts.^16^ Nanocatalyst is a collection of catalyst knowledge and nanotechnology. As the particle size decreases to the nanoscale, the available surface area increases. Therefore, nanocatalysts are the bridge between homogeneous and heterogeneous catalysts. On the one hand, nanocatalysts have high selectivity and efficiency similar to homogeneous catalysts. On the other hand, the heterogeneous nature of nanocatalysts makes them recyclable and reusable similar to heterogeneous catalysts.^17–24^ In this context, several nanostructure materials such as mesoporous materials,^25,26^ carbon nanostructures,^27,28^ modified polymers,^13,29,30^ modified graphene oxide,^31^ MOF structures,^32,33^ modified boehmite nanoparticles,^34,35^ biochar,^36^ magnetic nanoparticles^37,38^ have been employed as catalyst supports. Among them, biochar has unique features, which can be formed by the pyrolysis of organic waste, plant residues, tree bark, and animal manure.^39,40^ Biochar, in addition to being biocompatible, is very inexpensive and has no toxicity. Also, the surface of BNPs can be modified because its surface is covered with carbonyl, carboxylic acid, and hydroxyl groups, in which BNPs are suitable for the stabilization of various catalyst species.^8,41^ Due to this property of biochar, they can be used as a catalyst or support catalyst. Therefore, we are reporting 3-(sulfamic acid)-propyltriethoxysilane on biochar nanoparticles (SAPES@BNPs) as a practical, biocompatible and recyclable catalyst for the selective oxidation of sulfides to sulfoxides using H_2_O_2_ under solvent-free conditions as a green media. Because sulfoxides are important intermediates in the synthesis of organic and biologically active molecules and important reactants for oxygen transport. For example, the insecticides modafinil and omeprazole are two practical examples of the intermediates of these compounds in the chemical and pharmaceutical industries.^42,43^ Many sulfoxide compounds also have medicinal properties, such as penicillin as an antibiotic, nelfinavir as a potent HIV inhibitor, and kynureninase inhibitors.^43–47^ Also, the catalytic application of SAPES@BNPs was investigated in the multicomponent condensation of aldehydes, malononitrile and dimedone toward the synthesizing of tetrahydrobenzo[*b*]pyrans in water under mild and green conditions. Tetrahydrobenzo[*b*]pyrans are interesting organic compounds, which have many applications, such as antianaphylactic, antifungal, anticancer, antibacterial, anticoagulant, antioxidant, antiviral, antileishmanial, spasmolytic, and antiallergenic.^48–51^

## 2. Experimental

### 2.1. Preparation of the catalyst

**Scheme 1:**
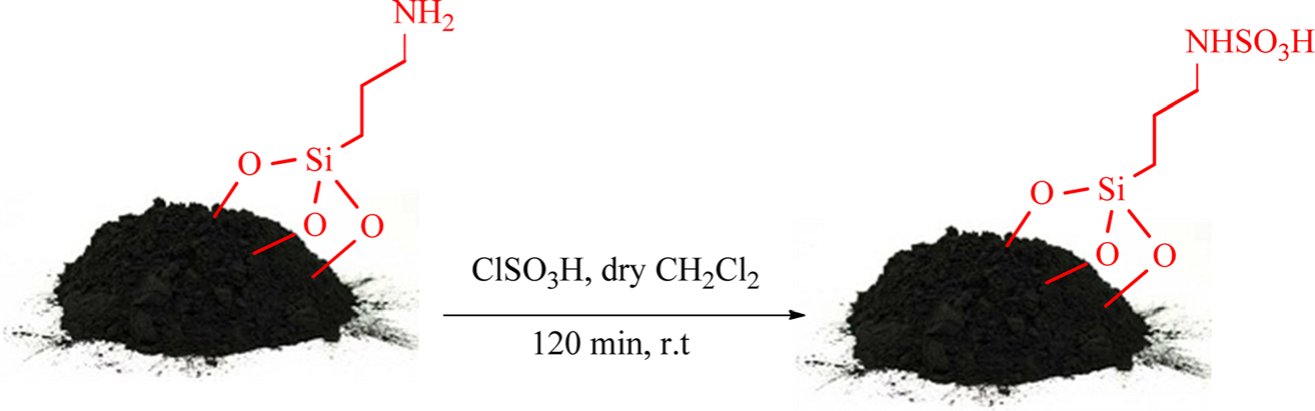
Schematic of the synthesis of SAPES@BNPs.

The modified biochar nanoparticles with (3-aminopropyl)triethoxysilane (APES@BNPs) were obtained based on authentic reported procedure.^41,52^ Then, in a 50 mL vacuum balloon, 0.5 g of APES@BNPs were dispersed in 3 mL of dry dichloromethane. Then, 0.8 mL of concentrated chlorosulfonic acid was added dropwise under a magnetic stirrer at room temperature. The resulting mixture was stirred under the same conditions for 120 min. The SAPES@BNPs catalyst was isolated by simple filtration. It was then washed twice with dichloromethane, twice with ethanol, and finally twice with dichloromethane. The resulting nanocatalyst was dried at room temperature (Scheme 1).

### 2.2. General method for the oxidation of sulfides to sulfoxides in the presence of SAPES@BNPs

**Scheme 2:**
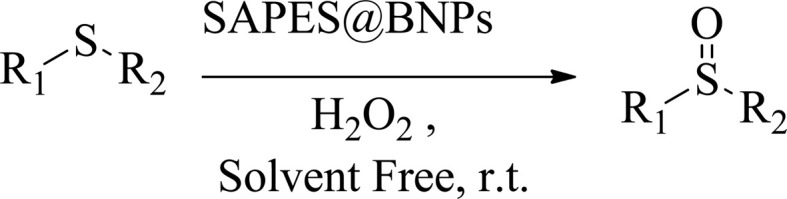
Oxidation of sulfides to sulfoxides in the presence of SAPES@BNPs nanocatalyst.

15 mg of SAPES@BNPs nanocatalyst was added to a mixture of sulfide (1 mmol) and hydrogen peroxide (0.5 mL). The reaction mixture was stirred under a solvent-free condition at room temperature for the periods listed in Table 2 with a magnetic stirrer. The reaction progress was followed by TLC paper using *n*-hexane/acetone solvent in a ratio of 8 : 2. Separation of the catalyst from the product was performed by simple filtration. The products were extracted with ethyl acetate and then the organic phase was dried over sodium sulfate. After evaporation of the solvent, the pure products were obtained with high yields (Scheme 2).

### 2.3. General method for the synthesis of tetrahydrobenzo[*b*]pyrans in the presence of SAPES@BNPs

**Scheme 3:**
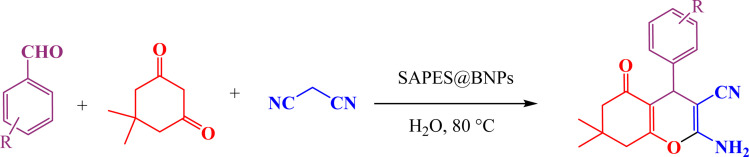
Preparation of tetrahydrobenzo[*b*]pyrans in the presence of SAPES@BNPs.

A multicomponent condensation of dimedone, malononitrile, and aldehyde derivatives was selected to prepare tetrahydrobenzo[*b*]pyrans (Scheme 3). Tetrahydrobenzo[*b*]pyrans were synthesized by stirring a mixture containing malononitrile (1 mmol), dimedone (1 mmol), and aldehyde derivatives (1 mmol) at 80 °C in H_2_O in the attendance of SAPES@BNPs (20 mg). The reaction proceed was supervised by TLC. Then, SAPES@BNPs were filtered, and the products were obtained in ethyl acetate. The obtained tetrahydrobenzo[*b*]pyrans were recrystallized in *n*-hexane.

### 2.4. Spectral data

#### 2.4.1 2-Amino-7,7-dimethyl-4-(2-nitrophenyl)-5-oxo-5,6,7,8-tetrahydro-4*H*-chromene-3-carbonitrile

^1^H NMR (250 MHz, DMSO): *δ*_H_ = 7.81–7.78 (d, *J* = 7.5 Hz, 1H), 7.67–7.61 (t, *J* = 7.5 Hz, 1H), 7.43–7.40 (d, *J* = 7.5 Hz, 1H), 7.37–7.32 (d, *J* = 5 Hz, 1H), 7.18 (br, 2H), 4.91 (s, 1H), 2.48 (s, 2H), 2.21–2.15 (d, *J* = 15 Hz, 1H), 2.02–1.96 (d, *J* = 15 Hz, 1H), 0.99 (s, 3H), 0.86 (s, 3H) ppm.

#### 2.4.2 2-Amino-7,7-dimethyl-4-(3-nitrophenyl)-5-oxo-5,6,7,8-tetrahydro-4*H*-chromene-3-carbonitrile

^1^H NMR (250 MHz, DMSO): *δ*_H_ = 8.07–8.04 (d, *J* = 7.5 Hz, 1H), 7.95 (s, 1H), 7.66–7.59 (t, *J* = 10 Hz, 2H), 7.16 (br, 2H), 4.39 (s, 1H), 2.52 (s, 2H), 2.28–2.22 (d, *J* = 15 Hz, 1H), 2.12–2.05 (d, *J* = 17.5 Hz, 1H), 1.02 (s, 3H), 0.93 (s, 3H) ppm.

#### 2.4.3 2-Amino-4-(3-hydroxyphenyl)-7,7-dimethyl-5-oxo-5,6,7,8-tetrahydro-4*H*-chromene-3-carbonitrile

^1^H NMR (250 MHz, DMSO): *δ*_H_ = 9.30 (br, 1H), 7.06–6.95 (m, 3H), 6.54–6.52 (m, 3H), 4.03 (s, 1H), 2.48 (s, 2H), 2.27–2.20 (d, *J* = 17.5 Hz, 1H), 2.11–2.04 (d, *J* = 17.5 Hz, 1H), 1.01 (s, 3H), 0.94 (s, 3H) ppm.

#### 2.4.4 2-Amino-4-(4-hydroxyphenyl)-7,7-dimethyl-5-oxo-5,6,7,8-tetrahydro-4*H*-chromene-3-carbonitrile

^1^H NMR (250 MHz, DMSO): *δ*_H_ = 9.23 (br, 1H), 6.91–6.88 (m, 4H), 6.64–6.61 (d, *J* = 7.5 Hz, 2H), 4.03 (s, 1H), 2.46 (s, 2H), 2.24–2.18 (d, *J* = 15 Hz, 1H), 2.09–2.02 (d, *J* = 17.5 Hz, 1H), 1.00 (s, 3H), 0.91 (s, 3H) ppm.

#### 2.4.5 (Sulfinylbis(methylene))dibenzene

^1^H NMR (250 MHz, CDCl_3_): *δ*_H_ = 7.37–7.30 (m, 10H), 4.44 (s, 2H), 4.19–4.13 (d, *J* = 15 Hz, 1H), 3.89–3.84 (d, *J* = 12.5 Hz, 1H) ppm.

#### 2.4.6 1-(Butylsulfinyl)butane

^1^H NMR (250 MHz, CDCl_3_): *δ*_H_ = 2.97–2.90 (t, *J* = 10 Hz, 4H), 1.86–1.74 (quin, *J* = 7.5 Hz, 4H), 1.53–1.39 (six, *J* = 7.5 Hz, 4H), 0.97–0.92 (t, *J* = 7.5 Hz, 6H) ppm.

#### 2.4.7 Tetrahydrothiophene 1-oxide

^1^H NMR (250 MHz, CDCl_3_): *δ*_H_ = 3.04–2.99 (t, *J* = 5 Hz, 4H), 2.24–2.15 (m, 4H) ppm.

## 3. Result and discussion

In this work, the characterization of SAPES@BNPs nanocatalyst was investigated using various techniques such as TGA, WDX, EDS, FTIR, SEM and XRD techniques.

**Fig. 1:**
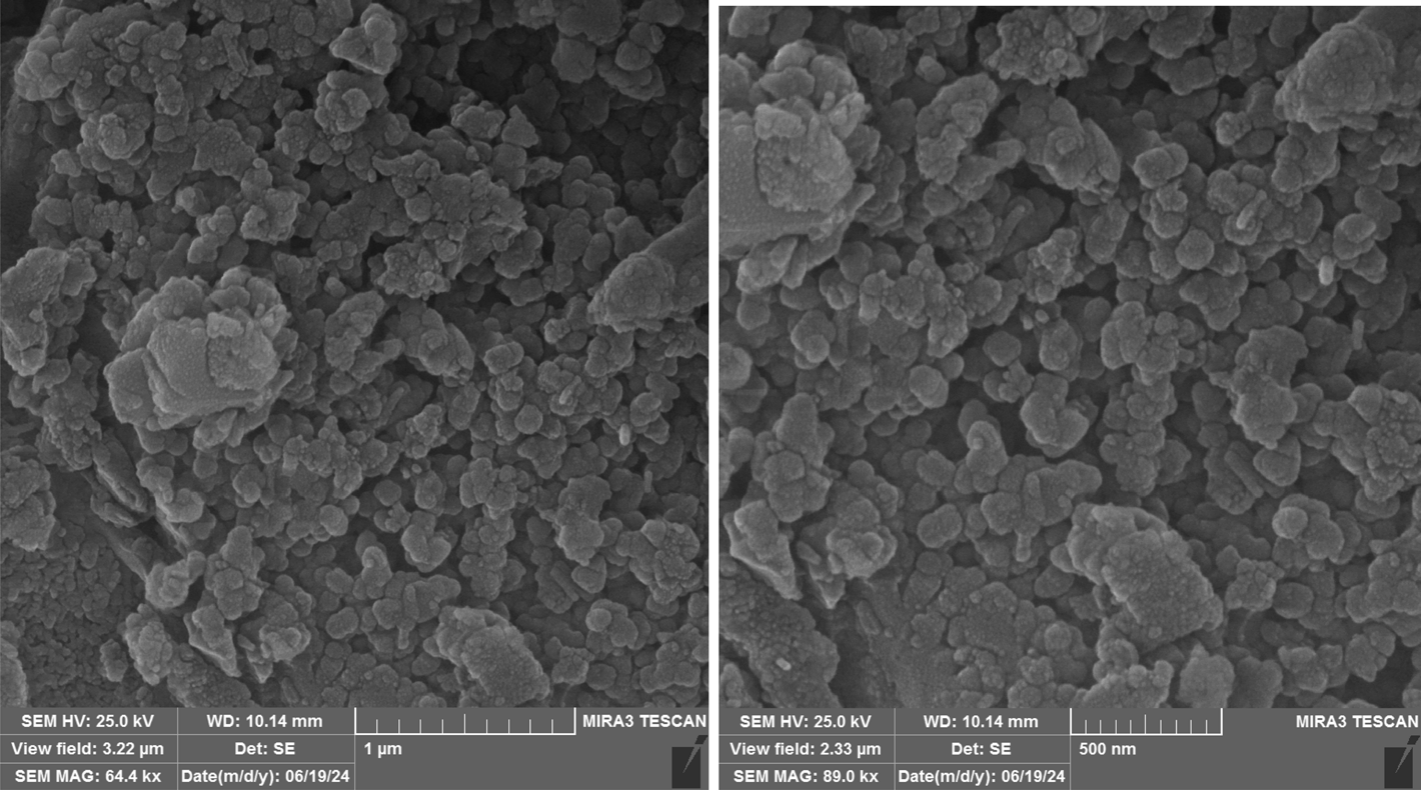
SEM images of biochar nanoparticles.

**Fig. 2:**
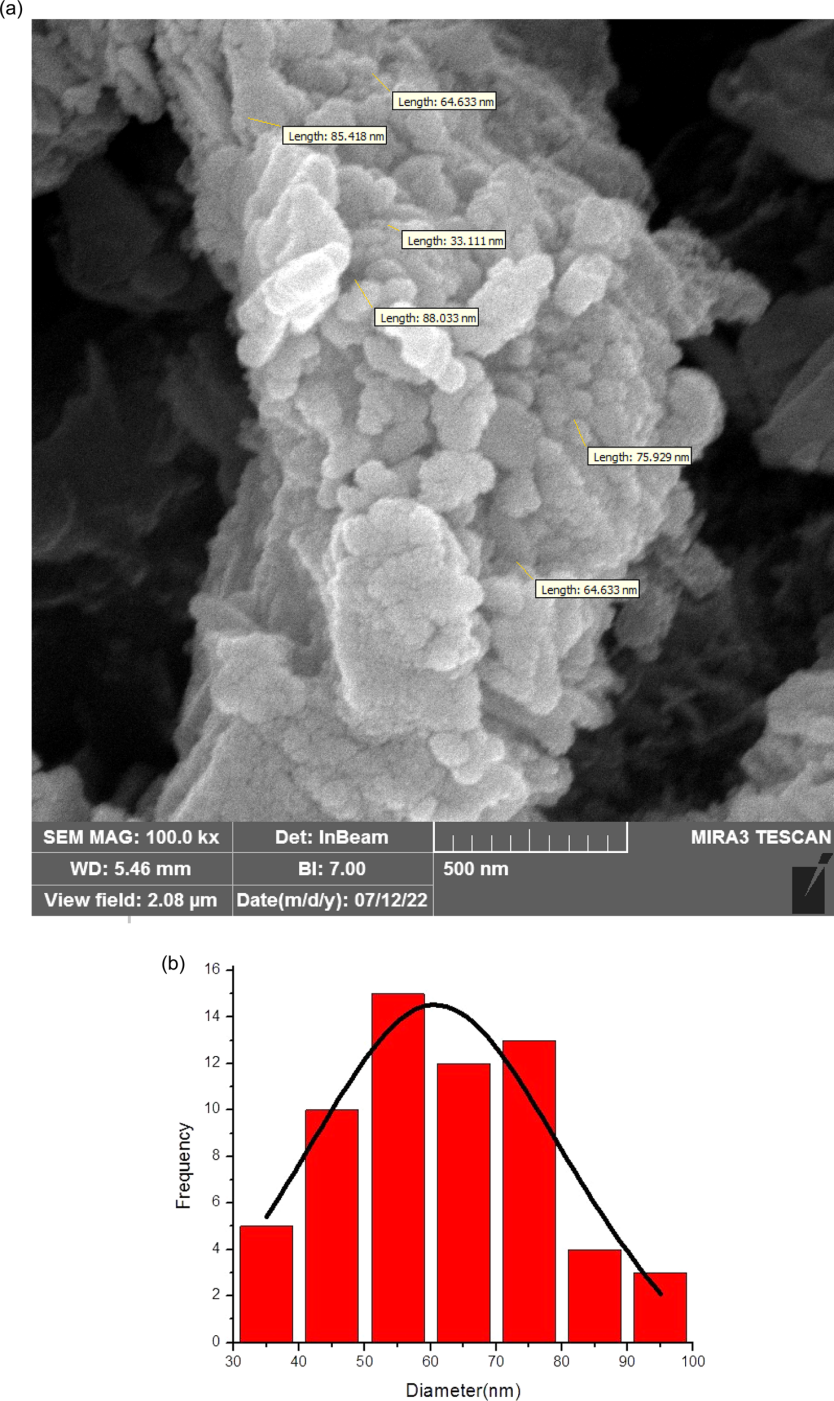
(a) SEM image of SAPES@BNPs and (b) particle size distribution histogram graphs of SAPES@BNPs particles, which was determined by SEM.

Fig. 1 shows the SEM images of biochar nanoparticles. Also, Fig. 2a shows the SEM image of SAPES@BNPs. As can be seen, the SEM images well demonstrated the quasi-spherical nature of SAPES@BNPs. The SEM technique was also used to determine the dimensions of the biochar nanoparticles and SAPES@BNPs. Therefore, there is no significant difference in the shape and size of biochar and SAPES@BNPs particles. The size of the particles is in the range of 30–100 nanometers (nm). Particle size distribution was obtained from the histogram graph from the SEM image (Fig. 2b). The histogram graph showed a large variation in the particle size of SAPES@BNPs. The particle size of SAPES@BNPs nanocatalyst was observed in the range of 30–100 nm with an average diameter size of about 60 nm.

**Fig. 3:**
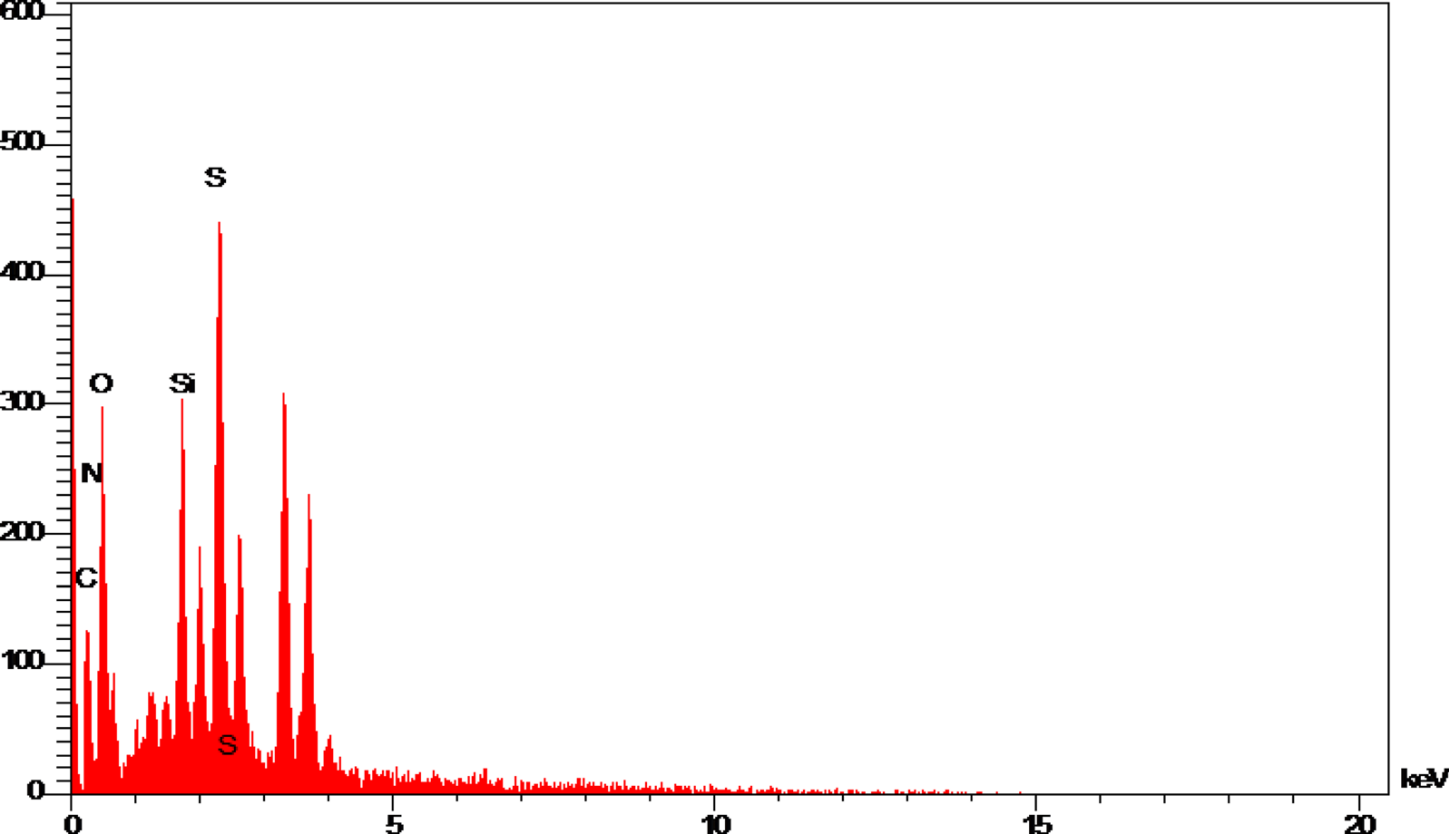
The EDS diagram of SAPES@BNPs.

X-ray energy diffraction technique (EDS) is a method to determine the elemental composition of a sample. In this work, EDS analysis was used to determine the quality of the elemental composition in SAPES@BNPs. EDS analysis of SAPES@BNPs is shown in Fig. 3 and all C, N, O, Si and S elements were observed in the catalyst structure.

**Fig. 4:**
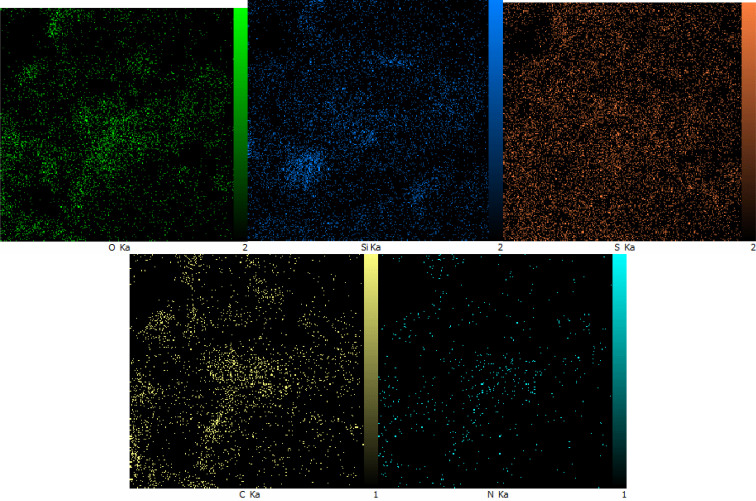
The WDX diagram of SAPES@BNPs.

Also, WDX (wavelength dispersive X-ray spectroscopy) was used to determine the distribution of elements in SAPES@BNPs. The WDX data on SAPES@BNPs nanocatalyst are shown in Fig. 4.

**Fig. 5:**
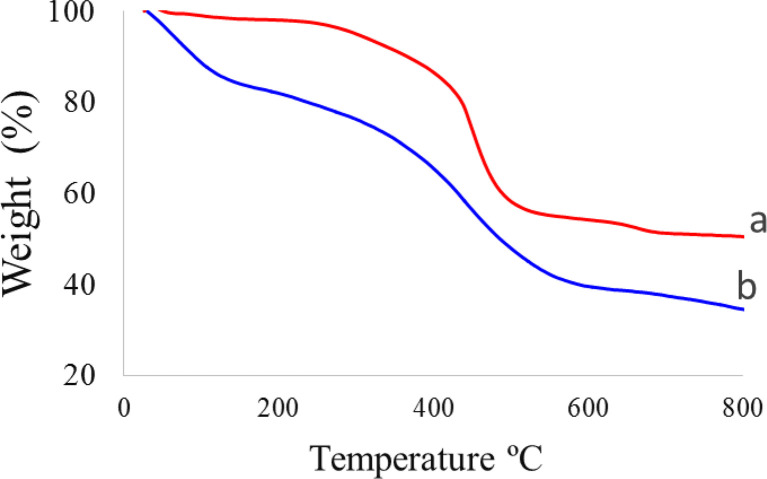
TGA diagrams of (a) biochar nanoparticles and (b) SAPES@BNPs nanocatalyst.

TGA analysis uses a specific heating program under a controlled atmosphere, in which weight changes of the samples are investigated. Based on the results of thermal gravimetric analysis (TGA), it is possible to estimate the amount of combustible or vaporizable materials, including water, other solvents and organic compounds in the sample. In this work, the TGA technique was used to determine the amount of organic materials that were stabilized on biochar nanoparticles (Fig. 5). The TGA diagrams of biochar nanoparticles and SAPES@BNPs nanocatalyst are shown in Fig. 5. The weight loss difference observed between biochar nanoparticles and SAPES@BNPs nanocatalyst was 16%, which meant that 16% of propyl sulfamic acid was immobilized on the surface of biochar nanoparticles. The weight loss of nearly 15%, which was observed in the first stage in the temperature range of less than 150 °C, is related to the evaporation of physically absorbed solvents and the removal of hydroxy groups attached to the biochar surface.^53^ In the second stage, a weight loss of about 45%, which was observed in the temperature range of 300 °C to 600 °C, is related to the removal of organic layers stabilized on the biochar.^19^ In the third stage, the weight loss of about 5%, which was observed in the temperature range above 600 °C, may be related to the continuation of pyrolysis of the biochar.^36^

**Fig. 6:**
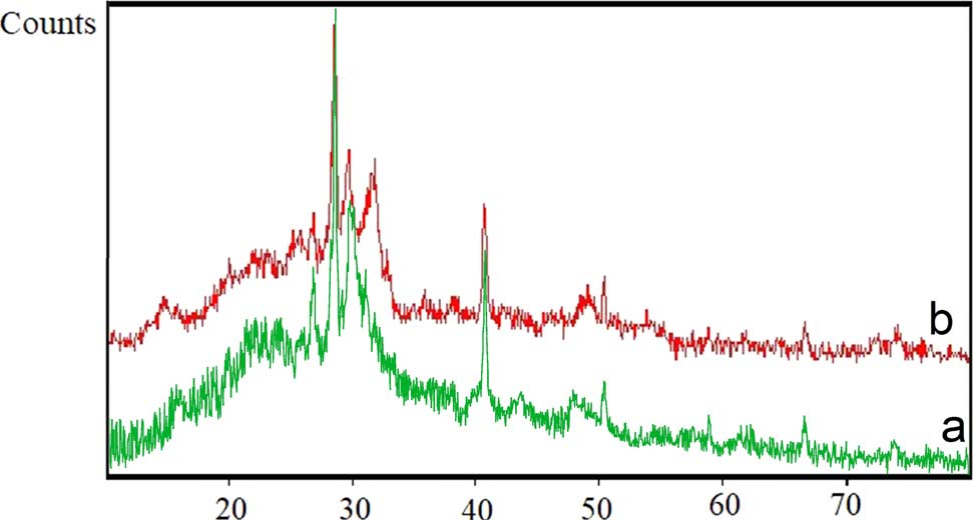
XRD patterns of (a) biochar nanoparticles and (b) SAPES@BNPs nanocatalyst.

XRD technique was used to investigate the structural order and determine the structural patterns of biochar nanoparticles and SAPES@BNPs. The obtained XRD patterns of biochar nanoparticles and SAPES@BNPs are shown in Fig. 6. A strong peak was observed in the region of 2*θ* = 30° for SAPES@BNPs, which is consistent with the structural pattern of biochar.^53^ Also, some weak peaks in the 2*θ* region for SAPES@BNPs can be seen at 40.7°, 43.7°, 48.7°, and 66.7°. As can be seen, the peaks of SAPES@BNPs are completely consistent with the structural pattern of biochar nanoparticles and this indicates the stability of the biochar after modification and immobilization of sulfamic acid.

**Fig. 7:**
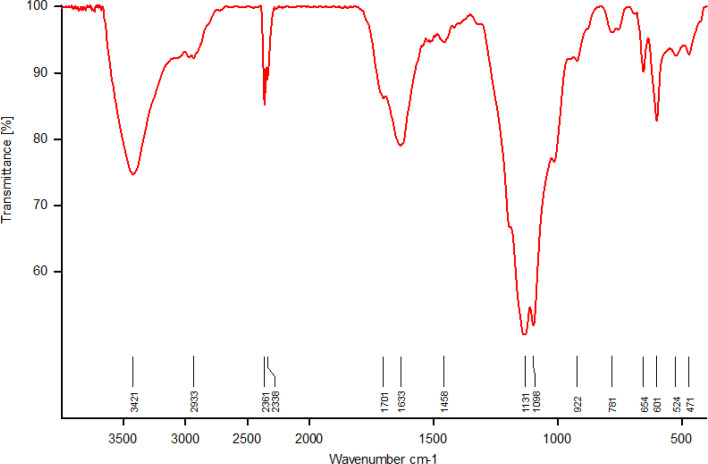
The FT-IR spectrum of SAPES@BNPs nanocatalyst.

The FT-IR spectrum of SAPES@BNPs is shown in Fig. 7. The strong bands at 1098, 781 and 471 cm^−1^ correspond to the vibrations of Si–O–Si bonds,^54^ indicating successful surface modification of biochar with (3-aminopropyl)triethoxysilane. A broad peak observed in the region of 2700–3700 cm^−1^ is related to the stretching vibration of sulfamic acid on biochar nanoparticles, which overlapped with the stretching vibrations of hydroxyl groups at 3421 cm^−1^ and C–H bonds at 2933 cm^−1^. Also, the stretching vibration of S–O was characterized by a band at 654 cm^−1^. A broad band at 1130–1250 cm^−1^ corresponds to the vibrations of S=O of sulfamic acid.

### 3.1. Catalytic study of SAPES@BNPs

At first, the catalytic application of SAPES@BN was investigated in the chemoselective oxidation of sulfides to sulfoxides.

The oxidation reaction of methyl phenyl sulfide was selected as an initial reaction for optimization of the reaction conditions, and the effect of the catalyst amount and solvent was investigated. This reaction did not progress in the absence of SAPES@BNPs catalyst, while, the best results were indicated when 20 mg of SAPES@BNPs catalyst was used. Then, the effect of the solvent was investigated. Among different solvents – *e.g.* water, ethyl acetate, *n*-hexane, dichloromethane, as well as the solvent-free conditions – the best result was indicated when the reaction was tested under solvent-free conditions (Table 1).

**Table 1:** The results of methyl phenyl sulfide oxidation in the presence of SAPES@BNPs using 0.5 mL of H_2_O_2_ at room temperature

Entry	Amount of the catalyst (mg)	Solvent	Time (min)	Yield (%)
1	—	Solvent-free	150	Trace
2	10	Solvent-free	120	65
3	20	Solvent-free	60	97
4	30	Solvent-free	45	97
5	20	CH_2_Cl_2_	60	46
6	20	*n*-Hexane	60	38
7	20	CH_3_COOEt	60	61
8	20	H_2_O	60	70

To expand the catalytic application of SAPES@BNPs, various sulfides – aliphatic sulfides and aromatic sulfides – were investigated under optimized conditions in the presence of the SAPES@BNPs catalyst. All sulfoxides were isolated in short reaction times with excellent yields (Table 2).

**Table 2:** The sulfoxidation of sulfides in the presence of SAPES@BNPs catalyst

Entry	Sulfide	Sulfoxide product	Time (min)	Yield (%)
1	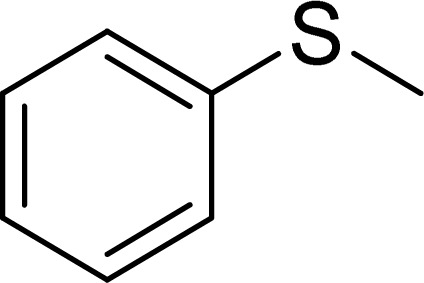	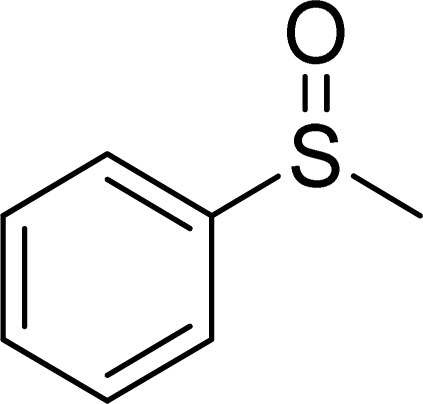	60	97
2	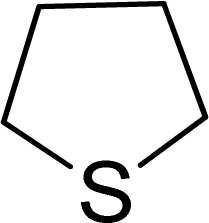	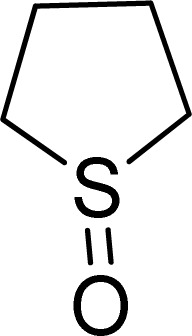	30	94
3	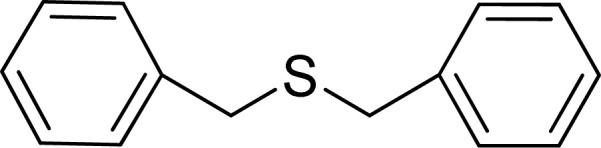	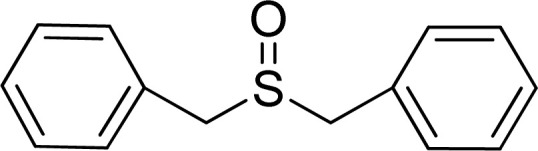	180	95
4	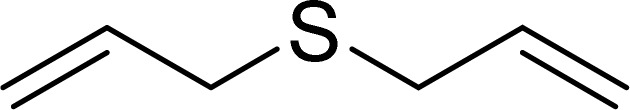	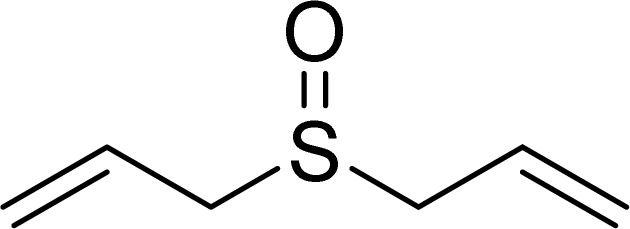	30	96
5	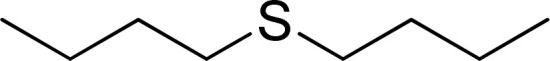	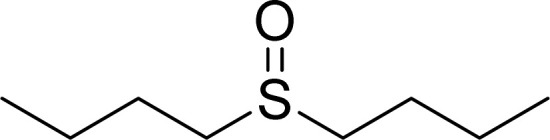	45	95

**Scheme 4:**
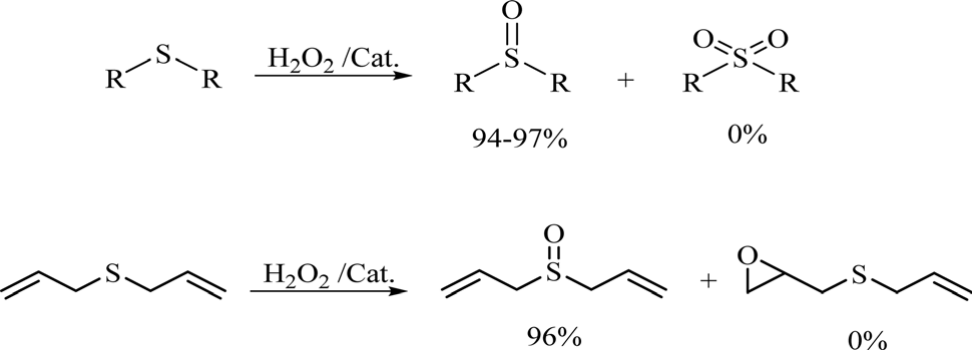
Chemoselectivity of SAPES@BNPs catalyst in the oxidation of sulfide.

One of the notable advantages and most important features of this work is the chemoselectivity of this system. The sulfoxidation of sulfides in the presence of SAPES@BNPs demonstrated good chemoselectivity (Scheme 4).

**Scheme 5:**
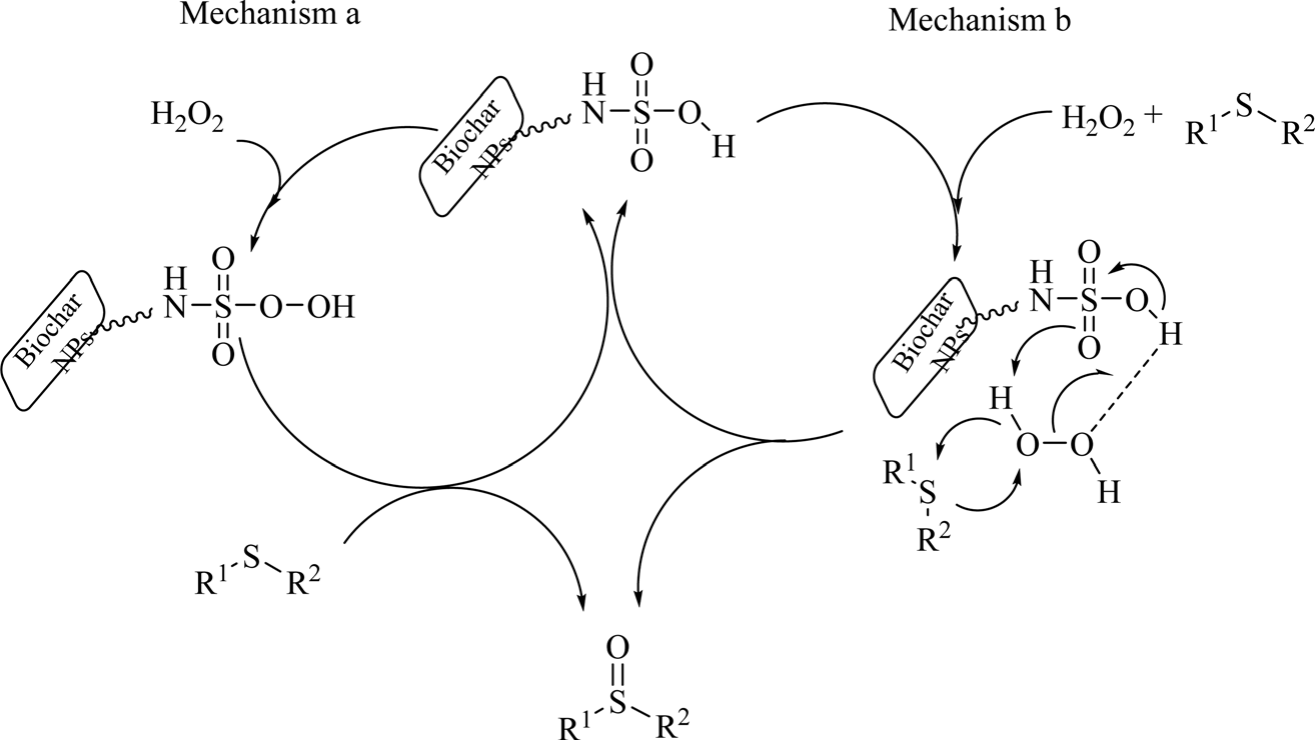
Suggested mechanism for the sulfoxidation of sulfides in the presence of SAPES@BNPs catalyst.

Based on past reports on the oxidation were sulfides,^55,56^ the following two mechanisms are suggested for the oxidation of sulfide in the presence of SAPES@BNPs catalyst (Scheme 5).

In the second part, the catalytic performance of SAPES@BNPs was investigated in the synthesis of tetrahydrobenzo[*b*]pyrans using condensation of aldehydes, malononitrile, and dimedone. In this regard, at first, the reaction conditions were optimized for the condensation of 4-chlorobenzaldehyde, malononitrile, and dimedone in terms of the nature solvent, amount of catalyst and the reaction temperature (Table 3). As it is clear in Table 3, the best result was obtained in the presence of 20 mg of SAPES@BNPs in water as solvent at 80 °C.

**Table 3:** Optimization conditions for the synthesis of tetrahydrobenzo[*b*]pyran using the SAPES@BNPs

Entry	Amount of the catalyst (mg)	Solvent	Temp (°C)	Time (min)	Yield (%)
1	15	H_2_O	80	17	90
2	20	H_2_O	80	10	96
3	25	H_2_O	80	5	97
4	20	H_2_O:PEG-400	80	10	31
5	20	H_2_O:EtOH	80	10	23
6	20	EtOH	80	10	15
7	20	H_2_O	60	45	91
8	25	H_2_O	r.t.	40	Trace

In continuation, we investigated different aldehydes to expand the scope catalytic activity of SAPES@BNPs for the synthesis of tetrahydrobenzo[*b*]pyran (Table 4). Substituted aldehydes with electron-donating or electron-withdrawing functional groups in ortho, meta, and para positions were investigated, and good results were obtained in all cases.

**Table 4:** Preparation of tetrahydrobenzo[*b*]pyran derivatives in the presence of SAPES@BNPs

Entry	Aldehyde	Product	Time (min)	Yield (%)
1	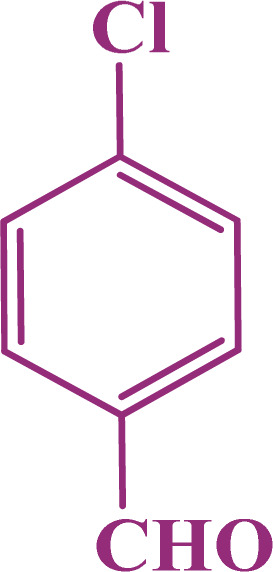	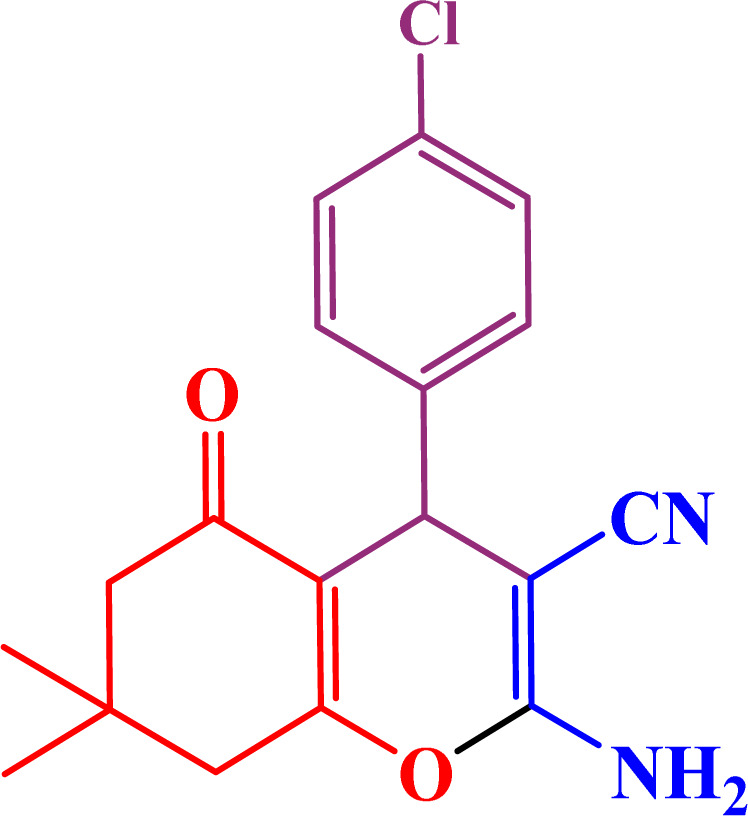	10	96
2	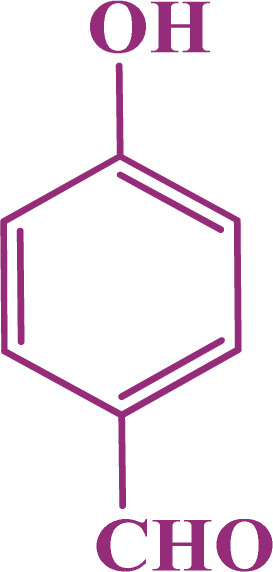	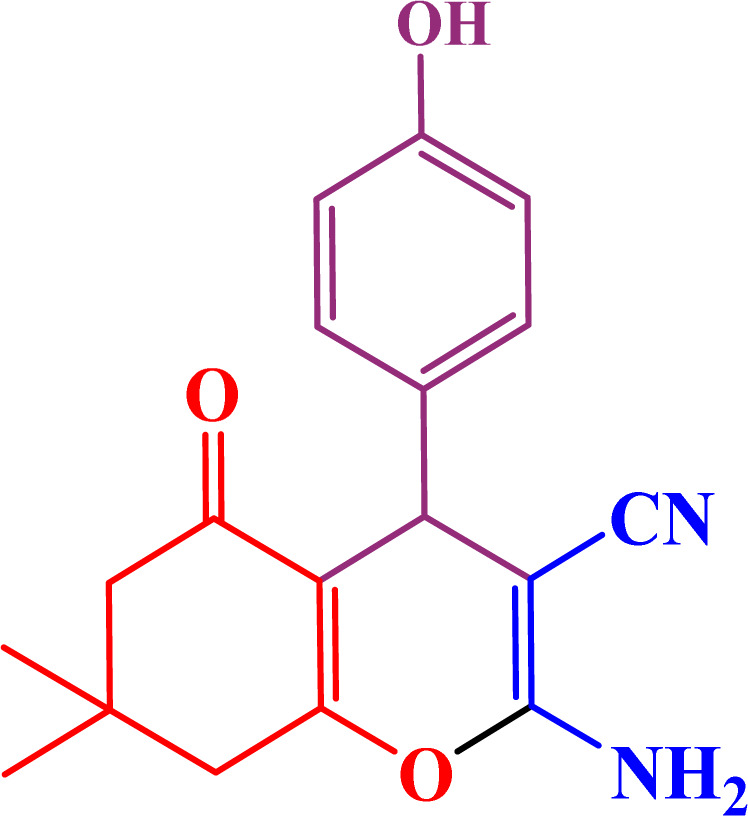	35	91
3	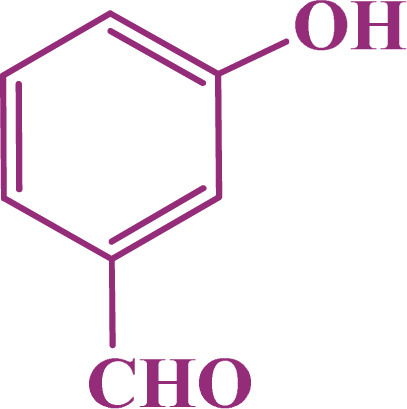	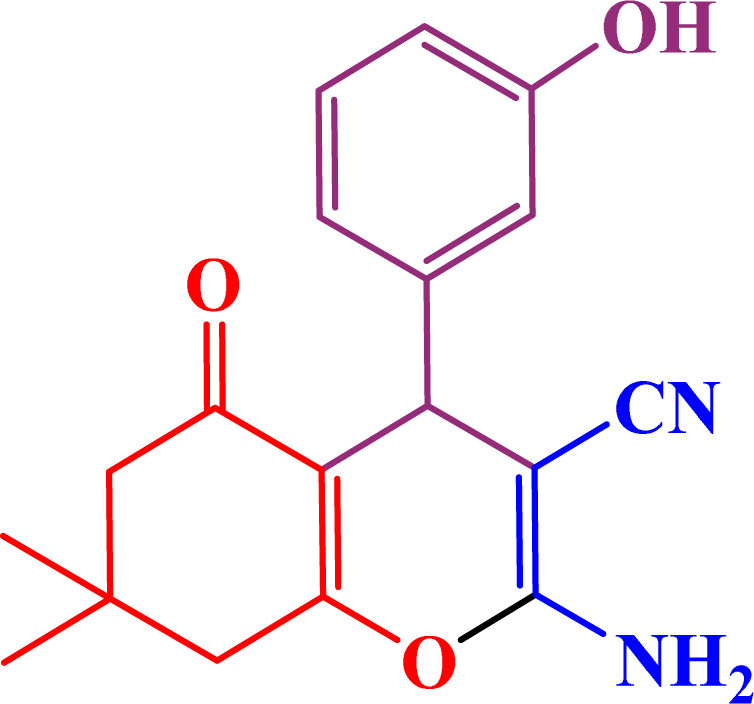	50	93
4	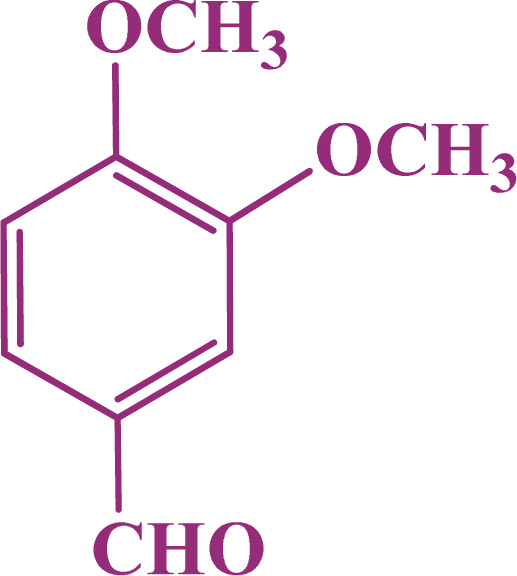	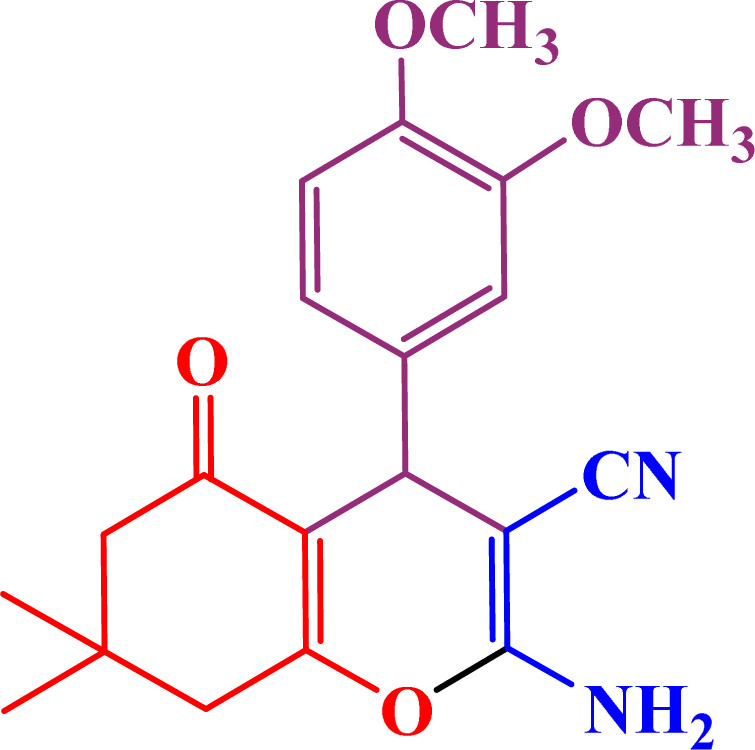	60	96
5	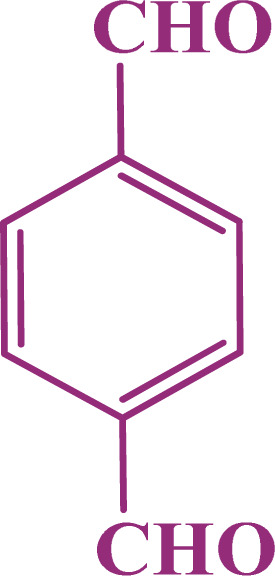	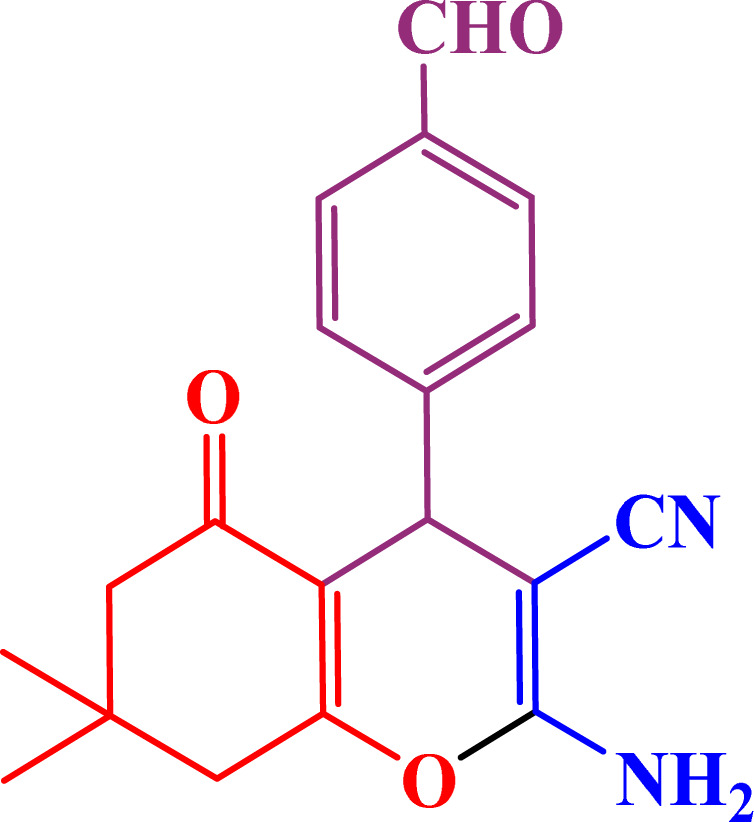	20	89
6	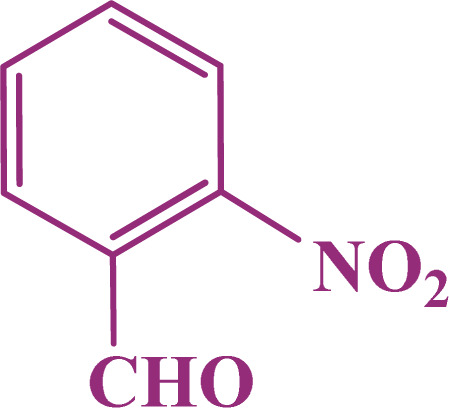	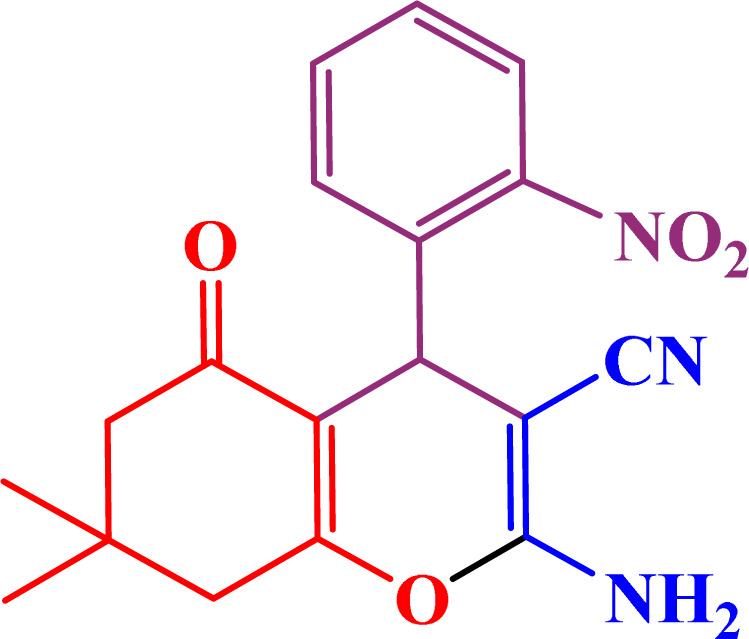	160	90
7	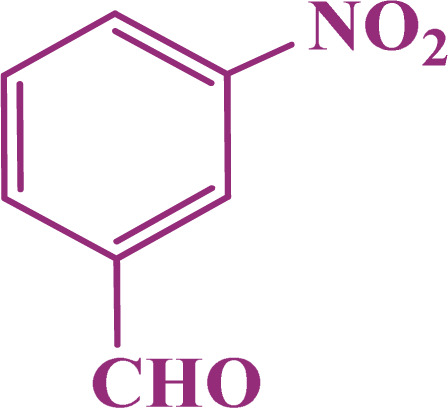	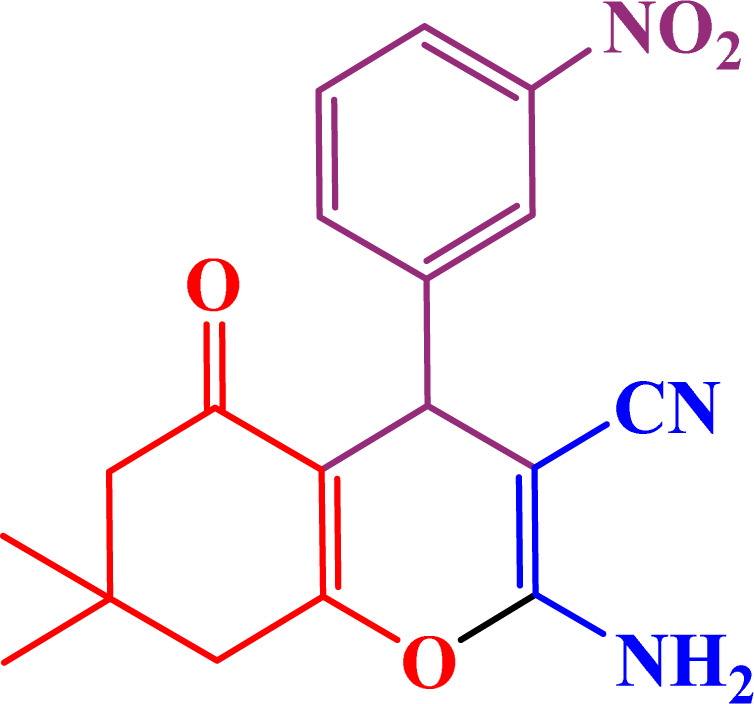	140	94
8	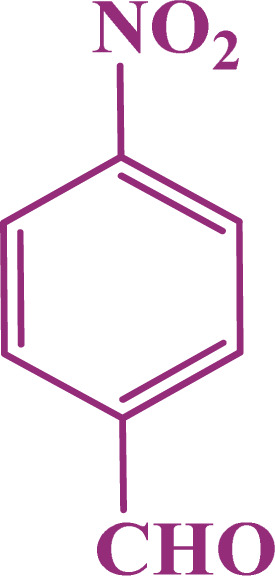	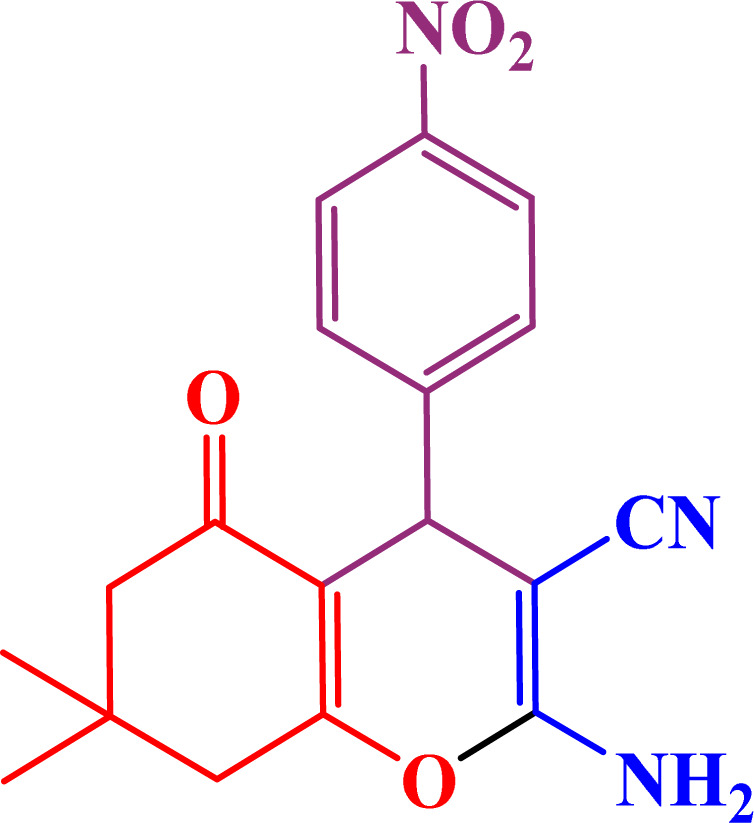	120	97

**Scheme 6:**
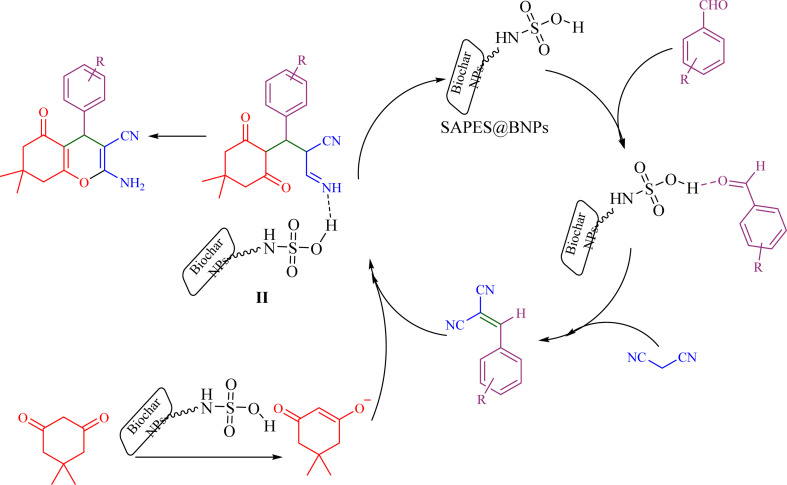
Suggested mechanism for the synthesis of tetrahydrobenzo[*b*]pyrans in the presence of SAPES@BNPs.

Based on the literature,^51^ a catalytic mechanism for the synthesis of tetrahydrobenzo[*b*]pyrans in the presence of SAPES@BNPs is shown in Scheme 6. At first, the carbonyl aldehyde group activated by SAPES@BNPs catalyst condenses with malononitrile through Knoevenagel-type condensation, which produces intermediate I. Dimedone also converts to enolic form in the presence of SAPES@BNPs, which is condensed with intermediate I to provide intermediate II. In the end, intermediate II is turned into the final product.

### 3.2. Recyclability of the catalyst

**Fig. 8:**
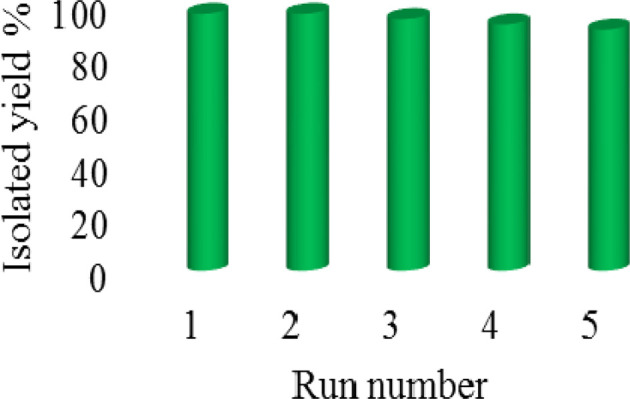
The recyclability of SAPES@BNPs in the oxidation of methyl phenyl sulfide.

Recyclability of the catalyst is the main advantage and important factor in heterogeneous systems. Therefore, the recyclability of SAPES@BNPs was investigated in the oxidation of methyl phenyl sulfide under defined conditions. In this investigation, the catalyst was isolated and then it was reused in the next run. As displayed in Fig. 8, the SAPES@BNPs catalyst can recycled up to 5 times at least.

### 3.3. Comparison of the catalyst

The practicability of SAPES@BNPs nanocatalyst in comparison with other reported catalysts in literature is illustrated in Table 5 in the oxidation of methyl phenyl sulfide. As displayed, SAPES@BNPs nanocatalyst provided 97% of (methylsulfinyl)benzene product within 1 h only, which is superior and better than other catalysts in terms of isolated yield of the product and the reaction time.

**Table 5:** Comparison of SAPES@BNPs nanocatalyst with other catalysts in the oxidation of methyl phenyl sulfide

Entry	Catalyst	Time (min)	Yield (%)	Ref.
1	V(O)-5NSA-MCM-41	300	97	43
2	Fe_3_O_4_@SiO_2_@l-arginine	120	96	45
3	Ni-DAMP-MOF	70	93	32
4	Ni-dithizone@boehmite	80	96	42
5	DSA@MNPs	360	98	56
6	Zr_6_-Irphen	360	98	57
7	Polyimide-P25	120	94	58
8	[VO(TPPABr)]CBr_3_	120	93	59
9	TsOH	240	88	60
10	Polymer-anchored Cu(ii)	180	90	61
11	VO_2_F(dmpz)_2_	300	95	62
12	SAPES@BNPs	60	97	This work

## 4. Conclusions

In this work, BNPs were first synthesized by pyrolysis progress of animal manure, and then, 3-(sulfamic acid)-propyltriethoxysilane was stabilized on its surface (SAPES@BNPs) as a heterogeneous acidic nanocatalyst. Then, the SAPES@BNPs catalyst was identified by various methods including TGA, XRD, SEM, EDS and WDX. The catalytic application of SAPES@BNPs was investigated in the chemoselective oxidation of sulfide to sulfoxide and the multicomponent synthesizing of tetrahydrobenzo[*b*]pyrans under mild and green conditions. One of the main advantages of this acid nanocatalyst is that it is affordable from an economic point of view because it is prepared from cheap and readily available raw materials.

## Data availability

All data are available in the “main manuscript” and “ESI”† files.

## Conflicts of interest

There are no conflicts to declare.

## Supplementary Material

RA-014-D4RA02265C-s001
