# Supplementary material for: 3-(Sulfamic acid)-propyltriethoxysilane on biochar nanoparticles as a practical, biocompatible, recyclable and chemoselective nanocatalyst in organic reactions
Source: RSC Adv. 2024 Jul 12;14(31):22147–58. doi: 10.1039/d4ra02265c (PMC11240877; doi:10.1039/d4ra02265c)

Electronic Supplementary Material (ESI) for RSC Advances.
This journal is © The Royal Society of Chemistry 2024

3-(sulfamic acid)-propyltriethoxysilane on biochar nanoparticles as a practical, biocompatibility, recyclable and chemoselective nanocatalyst in the organic reactions

Norolhoda Emad-Abbas[1], Jalil Naji[1,*], Parisa Moradi[2,*], Tavan Kikhavani[3]

[1]Department of Physics, Faculty of science, Ilam University, Ilam, Iran. E-mail: j.naji@ilam.ac.ir
[2] Department of Chemistry, Faculty of Science, Ilam University, P.O. Box 69315516, Ilam, Iran.
[3] Department of Chemical Engineering, Faculty of Engineering, Ilam University, Ilam, Iran.

Abstract

Recyclable and inexpensive catalysts, waste regeneration, utilize available and safe solvents are important principles of green chemistry. So in this project, biochar nanoparticles (BNPs) were synthesized by the pyrolysis method from chicken manure. Then, 3-(sulfamic acid)-propyltriethoxysilane (SAPES) was immobilized on the surface of BNPs (SAPES@BNPs). The prepared catalyst (SAPES@BNPs) was used in the selective oxidation of sulfides to sulfoxides as commercial, practical, biocompatibility and reusable catalyst. Also, the catalytic application of SAPES@BNPs was used in the multicomponent synthesizing of tetrahydrobenzo[b]pyrans under mild and green conditions. BNPs were characterized by SEM, TGA and XRD techniques. SAPES@BNPs were characterized by SEM, FT-IR, WDX, EDS, TGA, XRD techniques. Particle size distribution was obtained by histogram graph. SAPES@BNPs can be recovered and reused several times. The purity of the products was studied by NMR spectroscopy.

Keywords: Biochar nanoparticles, chemoselective catalyst, sulfamic acid, oxidation of sulfides, sulfoxides, tetrahydrobenzo[b]pyrans, multicomponent reactions.

Spectral data

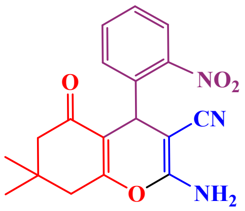

2-amino-7,7-dimethyl-4-(2-nitrophenyl)-5-oxo-5,6,7,8-tetrahydro-4H-chromene-3-carbonitrile: ^{1}H NMR (250 MHz, DMSO): δ_H= 7.81-7.78 (d, *J*= 7.5 Hz, 1H), 7.67-7.61 (t, *J*= 7.5 Hz, 1H), 7.43-7.40 (d, *J*= 7.5 Hz, 1H), 7.37-7.32 (d, *J*= 5 Hz, 1H), 7.18 (br, 2H), 4.91 (s, 1H), 2.48 (s, 2H), 2.21-2.15 (d, *J*= 15 Hz, 1H), 2.02-1.96 (d, *J*= 15 Hz, 1H), 0.99 (s, 3H), 0.86 (s, 3H) ppm.

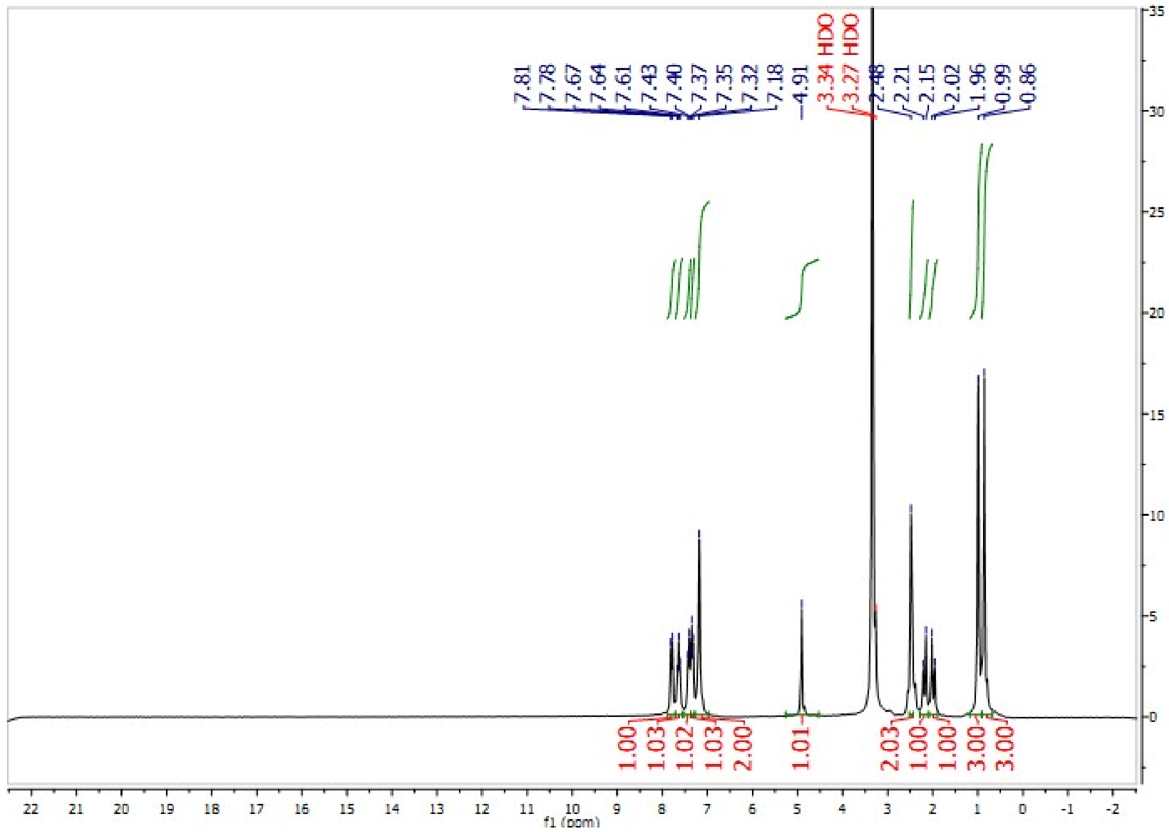

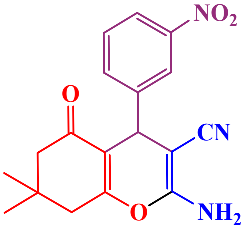

2-amino-7,7-dimethyl-4-(3-nitrophenyl)-5-oxo-5,6,7,8-tetrahydro-4H-chromene-3-carbonitrile: ^{1}H NMR (250 MHz, DMSO): δ_H= 8.07-8.04 (d, *J*= 7.5 Hz, 1H), 7.95 (s, 1H), 7.66-7.59 (t, *J*= 10 Hz, 2H), 7.16 (br, 2H), 4.39 (s, 1H), 2.52 (s, 2H), 2.28-2.22 (d, *J*= 15 Hz, 1H), 2.12-2.05 (d, *J*= 17.5 Hz, 1H), 1.02 (s, 3H), 0.93 (s, 3H) ppm.

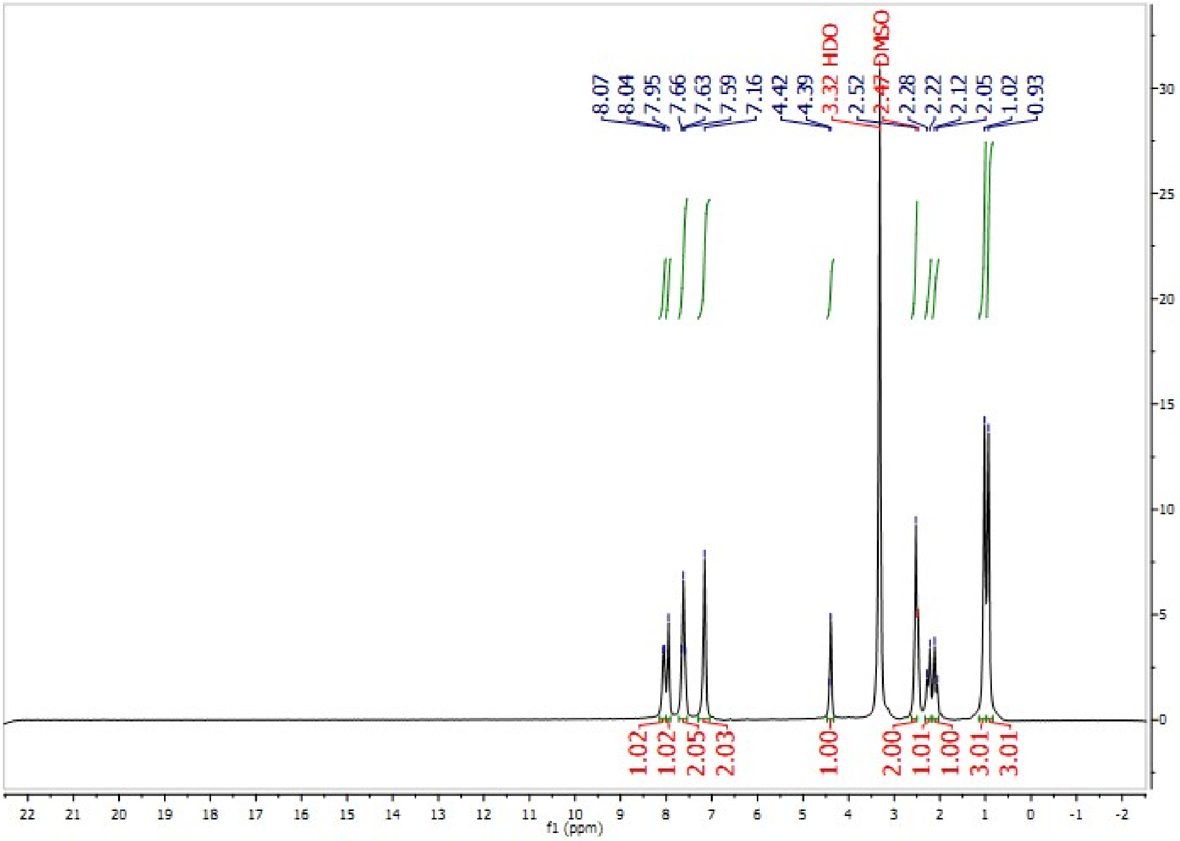

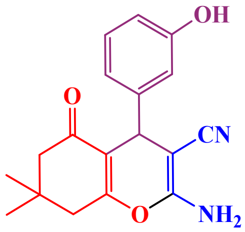

2-amino-4-(3-hydroxyphenyl)-7,7-dimethyl-5-oxo-5,6,7,8-tetrahydro-4H-chromene-3-carbonitrile:: ^{1}H NMR (250 MHz, DMSO): δ_{H}= 9.30 (br, 1H), 7.06-6.95 (m, 3H), 6.54-6.52 (m, 3H), 4.03 (s, 1H), 2.48 (s, 2H), 2.27-2.20 (d, *J*= 17.5 Hz, 1H), 2.11-2.04 (d, *J*= 17.5 Hz, 1H), 1.01 (s, 3H), 0.94 (s, 3H) ppm.

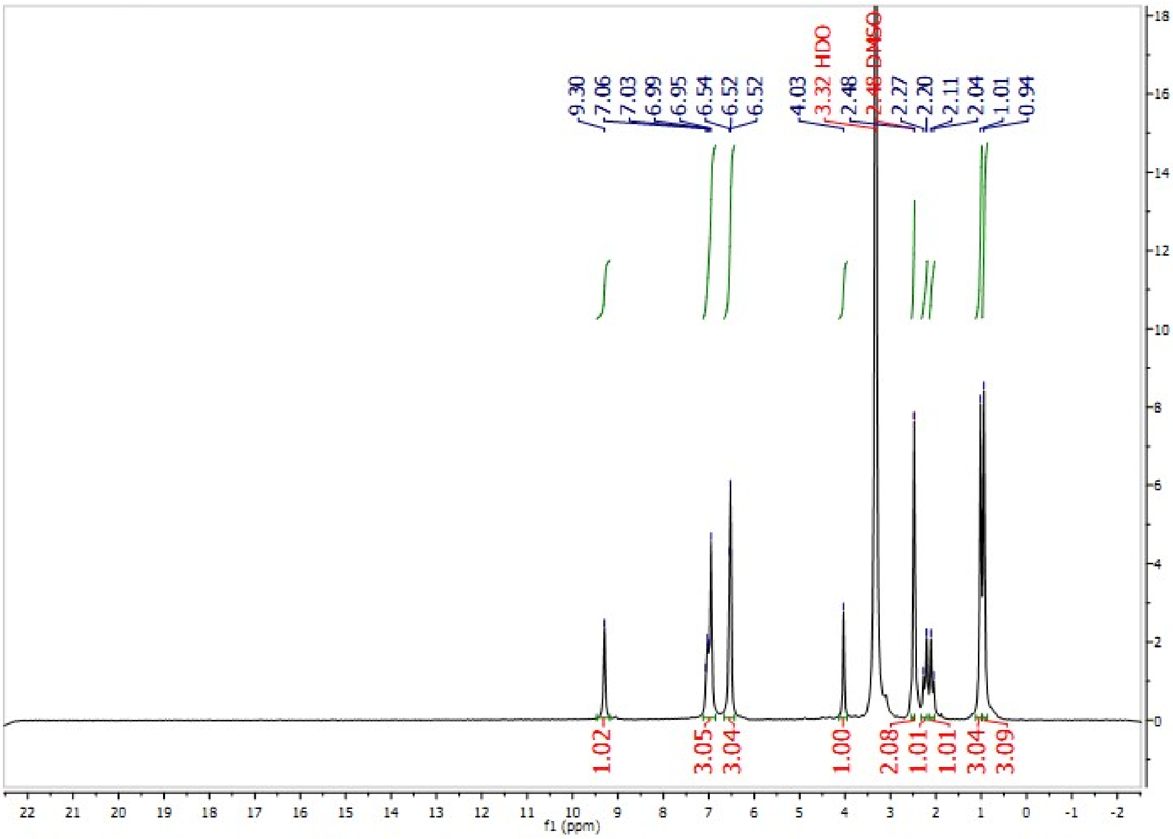

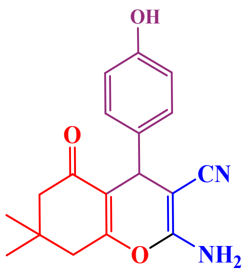

2-amino-4-(4-hydroxyphenyl)-7,7-dimethyl-5-oxo-5,6,7,8-tetrahydro-4H-chromene-3-carbonitrile: ^{1}H NMR (250 MHz, DMSO): δ_H= 9.23 (br, 1H), 6.91-6.88 (m, 4H), 6.64-6.61 (d, *J*= 7.5 Hz, 2H), 4.03 (s, 1H), 2.46 (s, 2H), 2.24-2.18 (d, *J*= 15 Hz, 1H), 2.09-2.02 (d, *J*= 17.5 Hz, 1H), 1.00 (s, 3H), 0.91 (s, 3H) ppm.

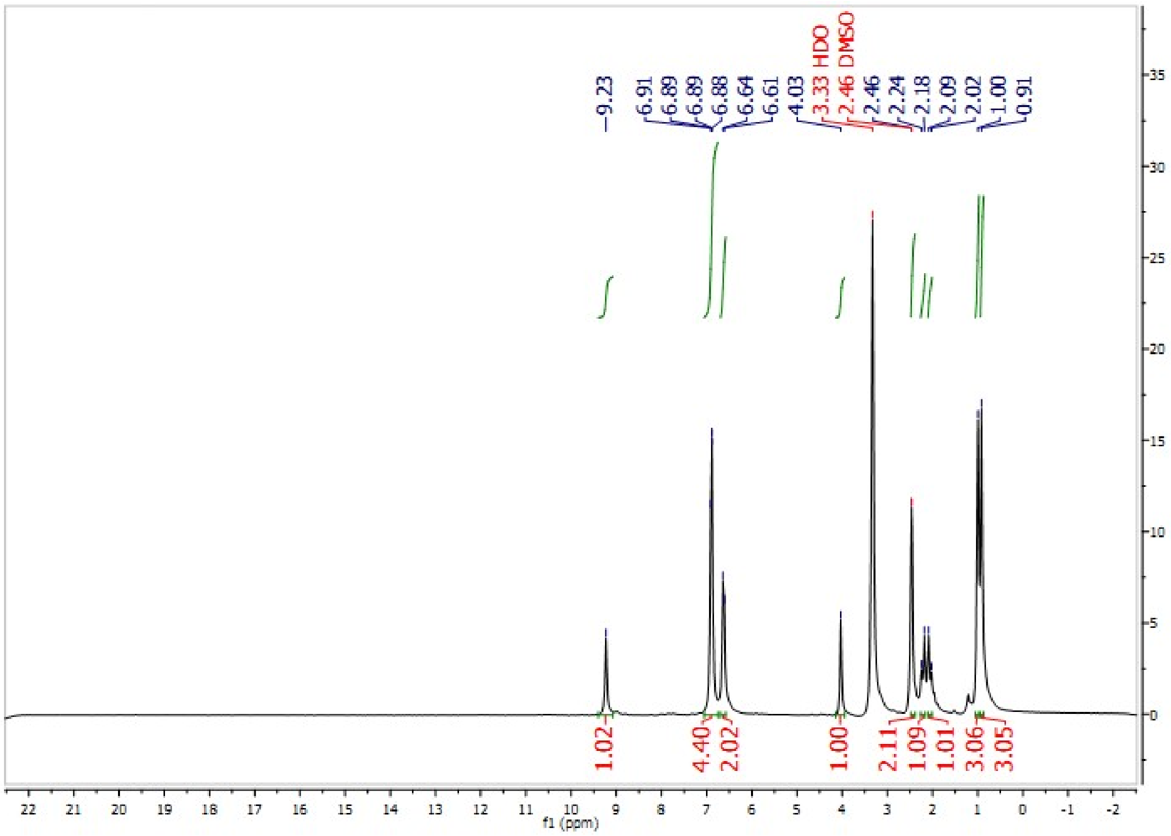

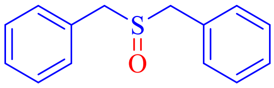

(Sulfinylbis(methylene))dibenzene: ^{1}H NMR (250 MHz, $CDCl_3$): δ_H= 7.37-7.30 (m, 10H), 4.44 (s, 2H), 4.19-4.13 (d, *J*= 15 Hz, 1H), 3.89-3.84 (d, *J*= 12.5 Hz, 1H) ppm.

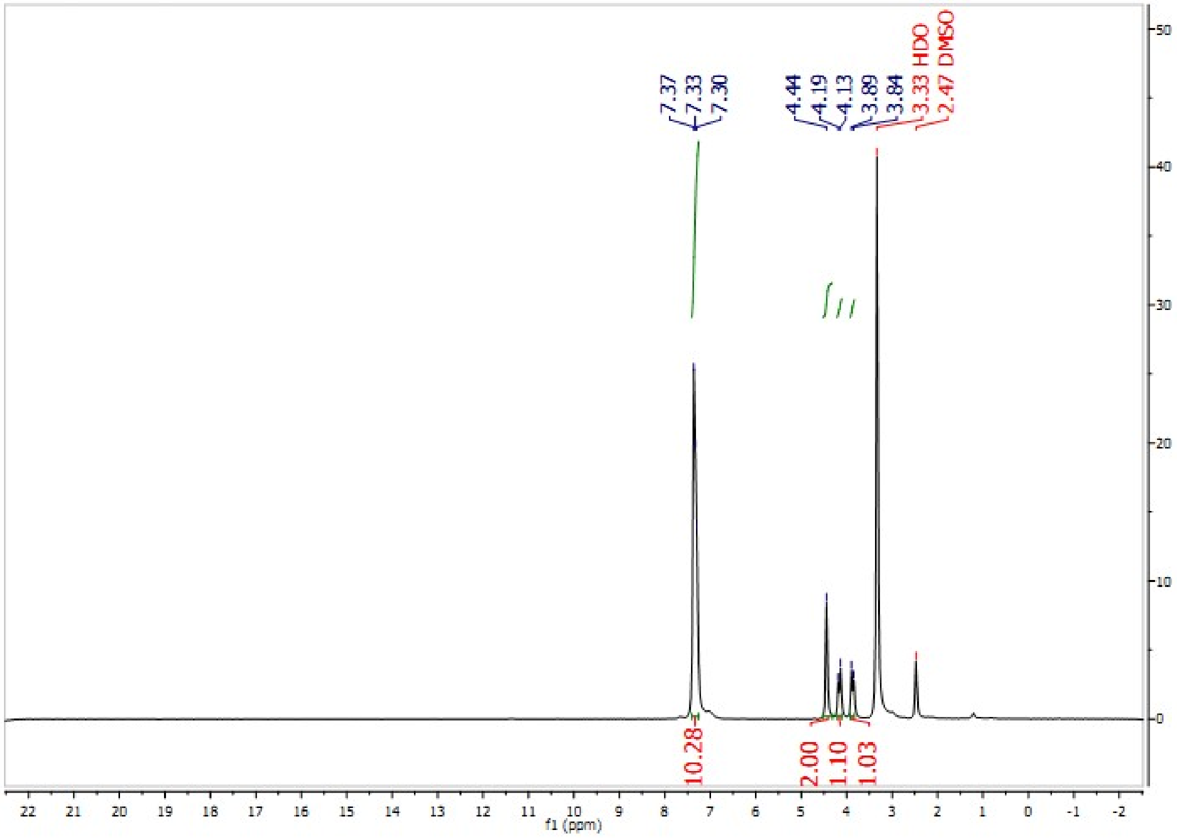

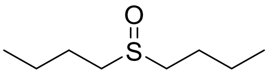

1-(butylsulfinyl)butane: ^{1}H NMR (250 MHz, $CDCl_3$): δ_H= 2.97-2.90 (t, *J*= 10 Hz, 4H), 1.86-1.74 (quin, *J*= 7.5 Hz, 4H), 1.53-1.39 (six, *J*= 7.5 Hz, 4H), 0.97-0.92 (t, *J*= 7.5 Hz, 6H) ppm.

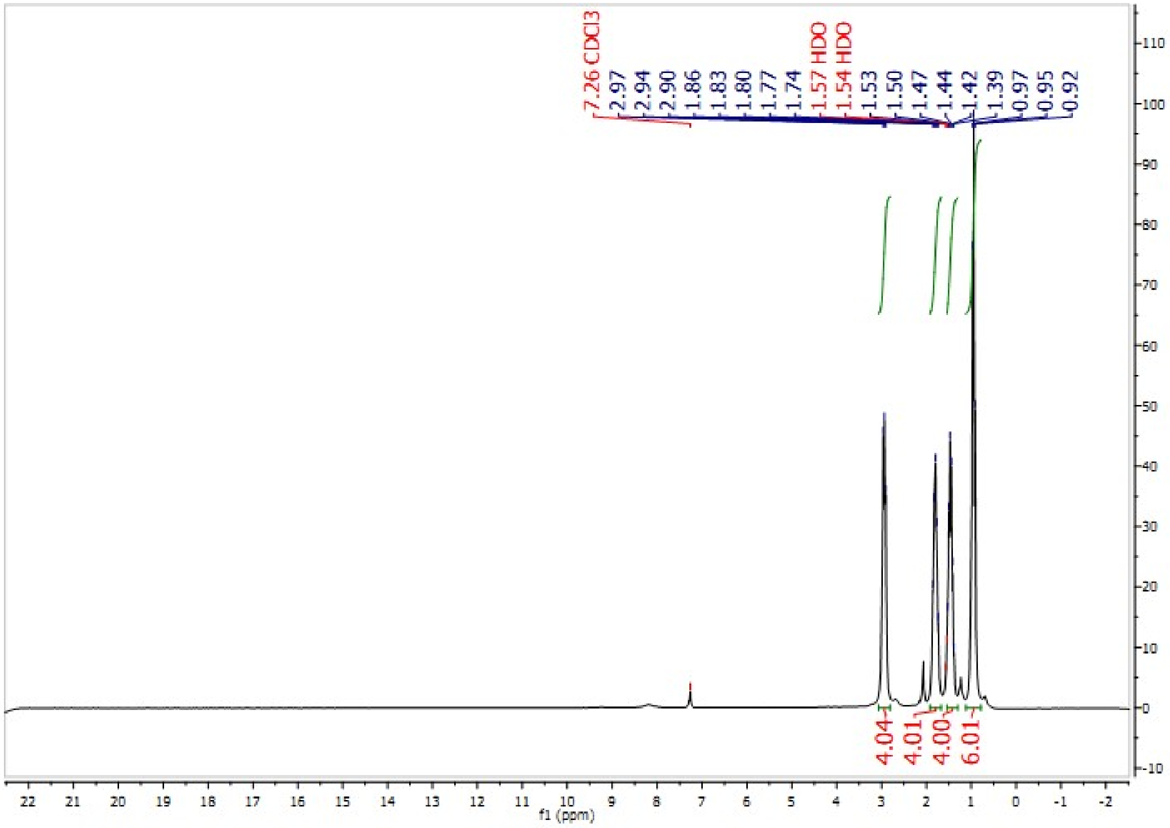

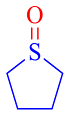

Tetrahydrothiophene 1-oxide: ^{1}H NMR (250 MHz, $CDCl_3$): δ_H= 3.04-2.99 (t, *J*= 5 Hz, 4H), 2.24-2.15 (m, 4H) ppm.

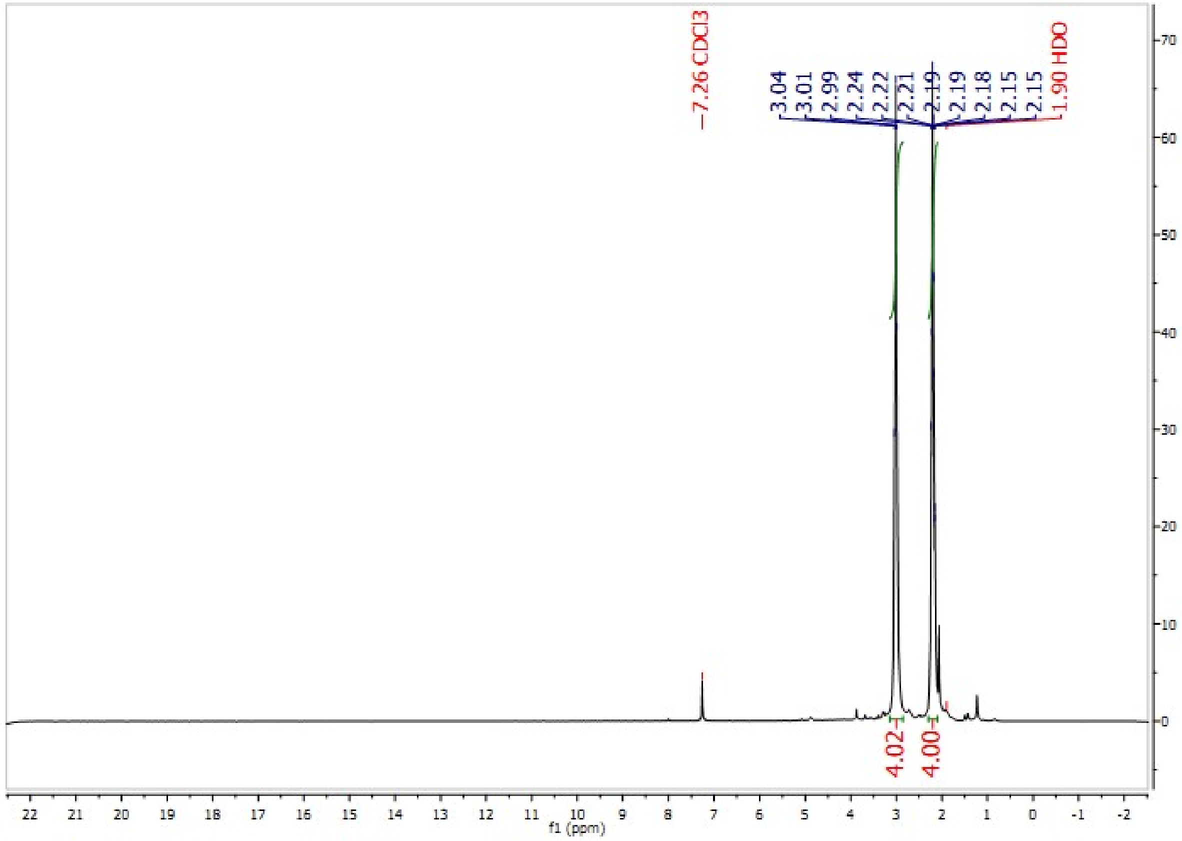

Supplement: RA-014-D4RA02265C-s001 [file RA-014-D4RA02265C-s001.pdf]
